# Thoracic ultrasound for diagnosing pneumopathies in neotropical primates

**DOI:** 10.3389/fvets.2024.1450104

**Published:** 2024-12-17

**Authors:** Jéssica Amancio Martins, Melina Castilho de Souza Balbueno, Soraya Kezam Málaga, Leonardo Dourado da Costa, Cidéli de Paula Coelho

**Affiliations:** ^1^University of Santo Amaro, São Paulo, Brazil; ^2^L&M Veterinária, Belo Horizonte, Brazil

**Keywords:** respiratory disease, *Callithrix* sp., lobar consolidation, lung, ultrasound

## Abstract

Lung ultrasound can be useful for the early diagnosis and treatment of respiratory complications. The combination of air and soft tissue confirms imaging artefacts that can contribute to differentiation between healthy and deteriorated lung tissue. Although non-human primates are often chosen as research models due to their anatomical and physiological similarity to humans, there is a lack of data on the use of lung ultrasound in these individuals. The aim of this study was to evaluate the contribution of ultrasound examinations of the thoracic region of *Callithrix* sp. for diagnosing pneumopathy. Parameters were obtained from 166 new world non-human primates of both sexes, aged between 1 and 15 years and weighing between 128 g and 680 g kept under human care at the Mucky Project in Itu, São Paulo. Thoracic ultrasound examinations were carried out using a LOGIQe—R7 device (GE, United States), with a 10–22 MHz linear transducer, at four points on the left and right antimeres. Among these 166 individuals, 72 had some kind of pulmonary alteration. Forty-one of the animals with pulmonary alterations diagnosed on ultrasound died and underwent necropsy. Histopathological examination showed that in half of the samples the lung tissue was compatible with some form of pneumopathy. Considering these cases, the pulmonary alterations diagnosed through thoracic ultrasound examination in *Callithrix* sp. can be correlated with the occurrence of pneumopathy, which is often asymptomatic. Lung ultrasound is an important tool for use in clinics to detect and monitor respiratory diseases and can save lives by enabling early treatment.

## 1 Introduction

Chest ultrasound examinations can be useful for the early detection and treatment of respiratory complications such as pneumonia, atelectasis and pleural effusion. They are of great value in both human and veterinary medicine, even in critically ill patients (1).

Non-human primates are widely used in anatomy and physiology research due to their similarities to humans, however, deforestation, habitat fragmentation, hunting for pets and hybridization are anthropogenic factors that can favour the population decline of NHP species, the lack of standardization in NHP species kept in captivity, makes it necessary to establish normal and pathological standards (2).

The lung parenchyma has a spongiform appearance. Its porosity and lung density result from a combination of elasticity and performance that allow remodeling of the air spaces, inflation, deflation, recruitment of peripheral air spaces and even thickening of the interalveolar septa (3). The bronchi, pulmonary bronchioles, ducts and alveolar sacs are components of the peripheral airspace. Their surface is protected by a thin continuous layer of surfactant, which is responsible for organizing the bubbles that fill the distal air spaces and provides structure and support, so that aeration of the lung parenchyma occurs properly (3, 4). Any pathological or functional situation that leads to a loss of porosity and lung density interferes with the relationship between air and tissue. This alteration can contribute to understanding lung ultrasound findings (3).

**Figure 1:**
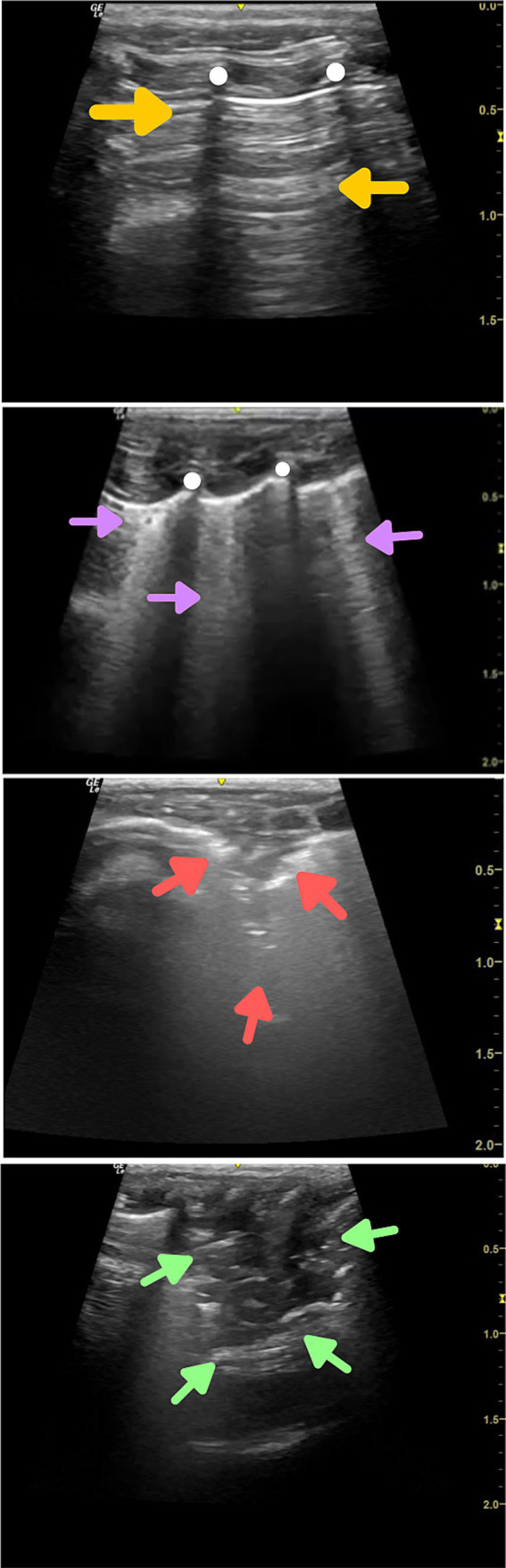
Thoracic ultrasound findings from *Callithrix* sp. **(A)** Healthy lung parenchyma, with the presence of parallel horizontal lines starting in the subpleural region and extending to the distal field (lines A—profile A) (yellow arrowheads). **(B)** Presence of parallel vertical hyperechoic artefacts starting in the subpleural region and extending to the distal field (lines B—profile B) (purple arrowheads). **(C)** Presence of consolidation with air bronchograms, represented by a hypoechoic area in the subpleural region, with poorly delimited outlines and the presence of vertical hyperechoic lines within the area described (profile C) (red arrowheads). **(D)** Presence of consolidation with static bronchogram, represented by a triangular-shaped hypoechogenic area, without vertical hyperechogenic lines inside the area described (green arrowheads).

In healthy lung tissue, there is a large amount of air and little water. The normal lung pattern is visualized as parallel lines in a horizontal direction starting in the subpleural region and extending to the distal field, in ultrasound this finding is denominated line A (Figure 1A) (3).

The thickened pulmonary septum located in the subpleural region has very small dimensions, making it impossible to visualize it on ultrasound (approximately 1 mm). However, when there is significant thickening, the beam becomes disorganized and this results in a difference in acoustic impedance in the image, compared with the adjacent area composed of air (5).

The liquid in the lung parenchyma produces an image of a small anechoic structure. Its resolution is lower than that of the ultrasound beam due to the difference in acoustic impedance caused by the presence of air. This causes reverberating vertical artefacts called B lines (Figure 1B), which would be found not under normal conditions but, rather, in alveolar-interstitial syndromes (5).

Air bronchograms are frequently seen on lung ultrasound and are characterized by hyperechoic spots or lines within the consolidated area. There are two types of air bronchogram: dynamic and static. Dynamic bronchograms are so called because they involve a centrifugal inspiratory movement that accompanies the patient’s respiratory movement. They are related to the irrigation capacity of the consolidated lung tissue through the airways and, for this reason, are often found in situations of pneumonia (Figure 1C). Static bronchograms are observed when there is no air inside the consolidation, due to a resorption process that contributes to reducing the volume of the consolidation (Figure 1D). This finding can be observed in late cases of atelectasis caused by reabsorption (6, 7).

Pulmonary consolidations are observed as delimited areas that resemble hepatic parenchyma (pulmonary hepatization), with well-defined regular or irregular outlines. Their acoustic impedance is high, due to the presence of multiple B lines or acoustic shadows over them (8).

Consolidations may be associated with alveolar impregnation caused by exudate, transudate, blood, fibrin or any other substance that replaces air (8). In the consolidated area, there is a loss of aeration, which forms a hypoechoic area located in the subpleural or tissue region (9).

Consolidations can be focal, partial or lobar (10). According to the appearance and location, it is possible to differentiate pneumonia from atelectasis due to tissue resorption. In patients with atelectasis due to tissue resorption, loss of pleural gliding is observed as an early sign, which differs from the presence of dynamic air bronchograms that glide in synchrony with respiratory movements. In 98.5% of cases, acute alveolar consolidations are in contact with the visceral pleural region. In late cases of atelectasis due to reabsorption, it will only be possible to visualize them when there is reabsorption of the gas content and a decrease in the volume of the consolidation, which is confirmed by a static bronchogram image (5).

When bronchopneumonia develops and compromises peripheral areas, there is a loss of aeration and the following ultrasound characteristics are observed: in patients with incipient infections involving interstitial inflammation, foci of B lines with irregular intervals are observed; and in cases of focal bronchopneumonia, small, consolidated areas are observed in the subpleural or lobar region, along with larger lobar consolidations. The presence of secretions and air in the bronchial region is observed on ultrasound as hyperechoic lines within the consolidated area, which move along in synchrony with breathing (5, 11).

The aim of this study was to assess the contribution of ultrasound examination of the thoracic region of individuals of *Callithrix* sp. to making the diagnosis of pulmonary abnormalities.

## 2 Methodology

All the procedures described in this study were authorized by the Ethics Committee for the Use of Animals (CEUA) at the University of Santo Amaro, Brazil, and the project was filed under the number 57/2021. The project was also authorized by SISBIO (Authorization for Activities for Scientific Purposes of the Brazilian Ministry of the Environment) under the number 78874-1. The letter of consent to carry out this study was then given to and countersigned by the person in charge of the Mucky Project, where the animals were living. The methodology involved evaluating the influence of factors such as sex, age, and body mass on ultrasound findings in marmosets, as well as statistically comparing the frequency of key findings between different species.

Thoracic ultrasonogram examinations were carried out using a LOGIQe - R7 device (GE, United States), with a 10–22 MHz linear transducer and with the aid of an acoustic gel. Images were obtained through the left and right parasternal windows.

**Figure 2:**
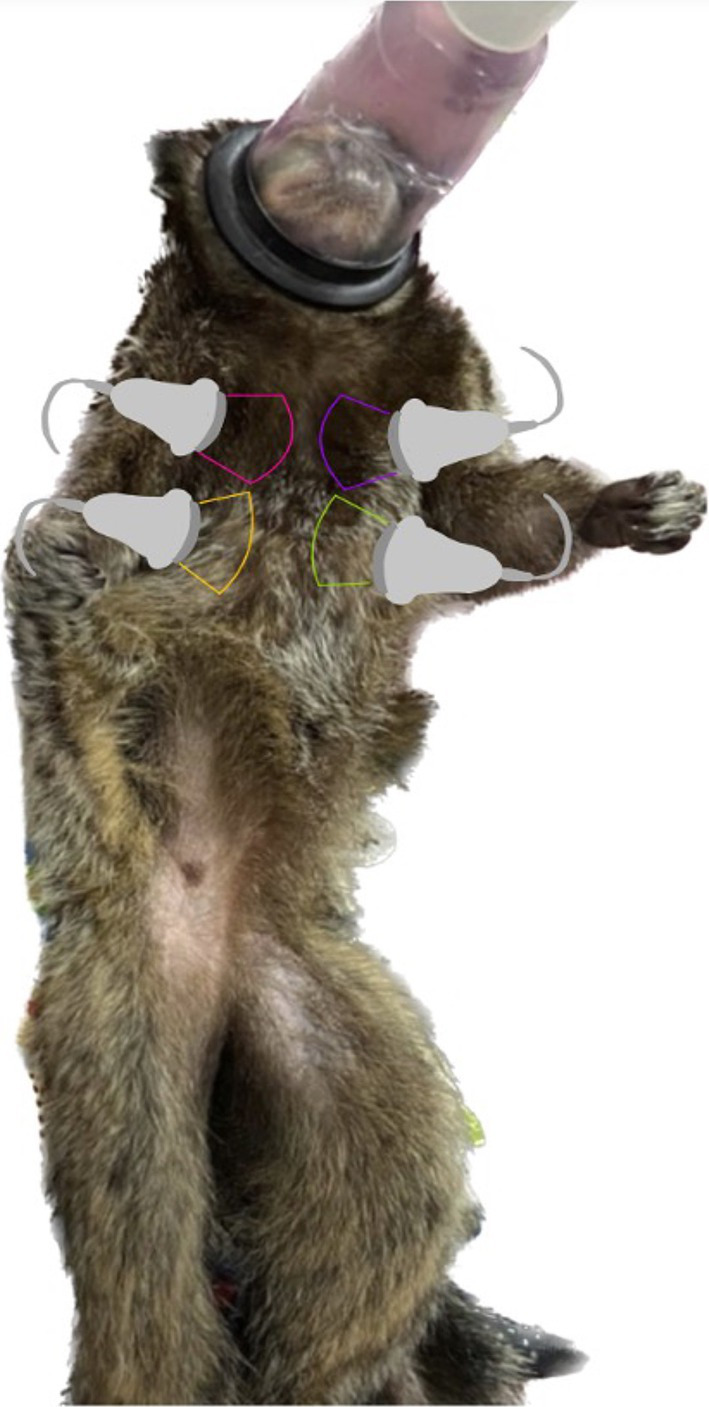
Image showing the points on *Callithrix* sp. that were assessed using the thoracic transducer.

The patients were sedated with isoflurane, induced via mask, and this was maintained at a rate of 1 to 3%, with 100% oxygen, for the duration of the examination, which lasted a maximum of 20 min (12). Before the examination, a four-hour water-and-food fast was imposed. The animals remained in a supine position during the examination and were assessed at four points on each antimere (Figure 2).

Thoracic sonographic findings were compared with histopathological results obtained from 10 animals that died during the study period.

## 3 Results

This observational study used a convenience sample made up of 166 individuals of *Callithrix* sp. (males and females), aged between 1 and 15 years (5.32 ± 3.76 years) and with an average weight of 128 g to 680 g (0.33 ± 0.07 kg). They were living within the premises of the NGO Mucky Project, in the municipality of Itu, São Paulo, Brazil, and the assessments were carried out between November 2, 2021, and January 24, 2022.

As regards the gender of the animals: 20 (12.04%) were *C. aurita* species [13 (65%) males and 7 (35%) females], 69 (41.5%) were *C. jacchus* species [34 (49.27%) males and 35 (50.72%) females], 30 (18.07%) were of the *C. penicilatta* species [13 (18.07%) males and 35 (50.72%) females] and 47 (28.31%) were *Callithrix* sp. hybrids [28 (59.57%) males and 19 (40.42%) females].

We sampled 166 marmosets (*Callithrix* spp.) with a mass of 0.33 ± 0.07 kg (0.13–0.68) and an age of 5.39 ± 3.21 years (0.70–15.00).

The species did not differ significantly in terms of mass (*H* = 5.24, df = 3, *p* = 0.155), but they did in terms of age (*H* = 43.69, df = 3, *p* < 0.001).

The sex ratio was 53% males to 47% females, which did not differ significantly from an equal sex ratio (*z* = −0.774, *p* = 0.439).

**Figure 3:**
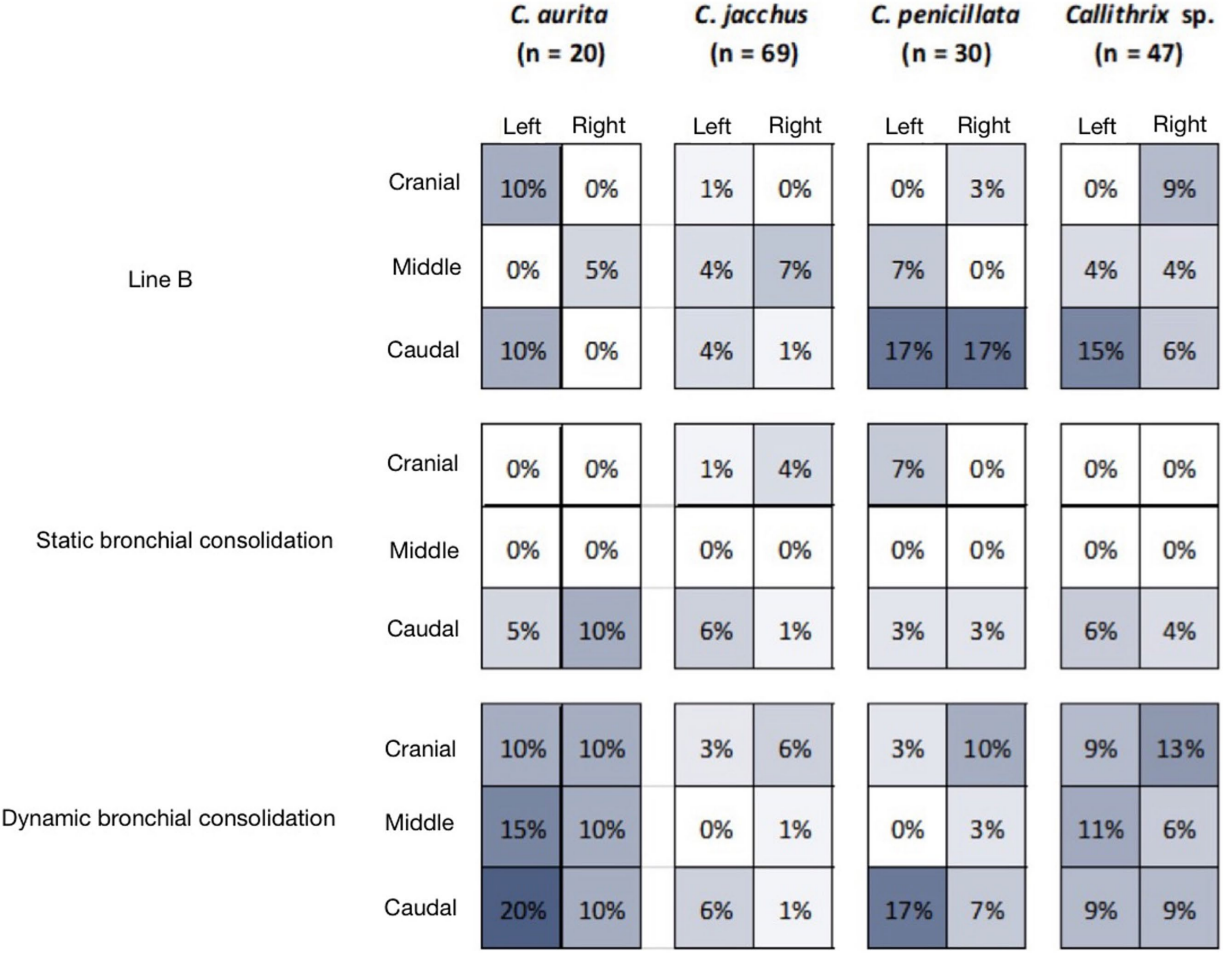
Visual representation of the frequency of anatomical distribution of ultrasound findings from the thorax of neotropical primates of the genus *Callithrix* sp.

Among the 166 individuals assessed, 43 (25.90%) had B lines, located as follows, 20 (12.04%) had a static bronchogram (SBC) and 43 (25.90%) had dynamic bronchograms (DBC) (Table 1). The distribution of the lesions is shown in a mosaic (Figure 3).

**Table 1:** Frequency of ultrasound findings (number of animals and percentage per species) from the thorax of neotropical primates of the genus *Callithrix*.

Taxon	Line A	Line B	SBC	DBC	*N*
*Callithrix aurita*	20 (100%)	5 (25%)	2 (10%)	7 (35%)	20
*Callithrix jacchus*	69 (100%)	12 (17%)	9 (13%)	12 (17%)	69
*Callithrix penicillata*	30 (100%)	11 (37%)	4 (13%)	10 (33%)	30
*Callithrix* sp.	47 (100%)	15 (32%)	5 (11%)	14 (30%)	47
Total	166 (100%)	43 (26%)	20 (12%)	43 (26%)	166

Source: The author, 2022.

Regarding concomitant findings, 30 animals had more than one type of pulmonary artefact, the occurrence of B-lines associated with consolidation, together with a dynamic bronchogram, was observed in 17 animals, with higher prevalence in the left caudal window, in 8 animals B-lines associated with consolidation, together with a static bronchogram, with higher prevalence in the left caudal window, in 1 animal B-lines associated with consolidation, together with a static bronchogram, and consolidation together with a dynamic bronchogram, the occurrence of consolidation with a static bronchogram, in association with consolidation with a dynamic bronchogram, was observed in 4 animals, with higher prevalence in the right cranial and right caudal windows.

**Figure 4:**
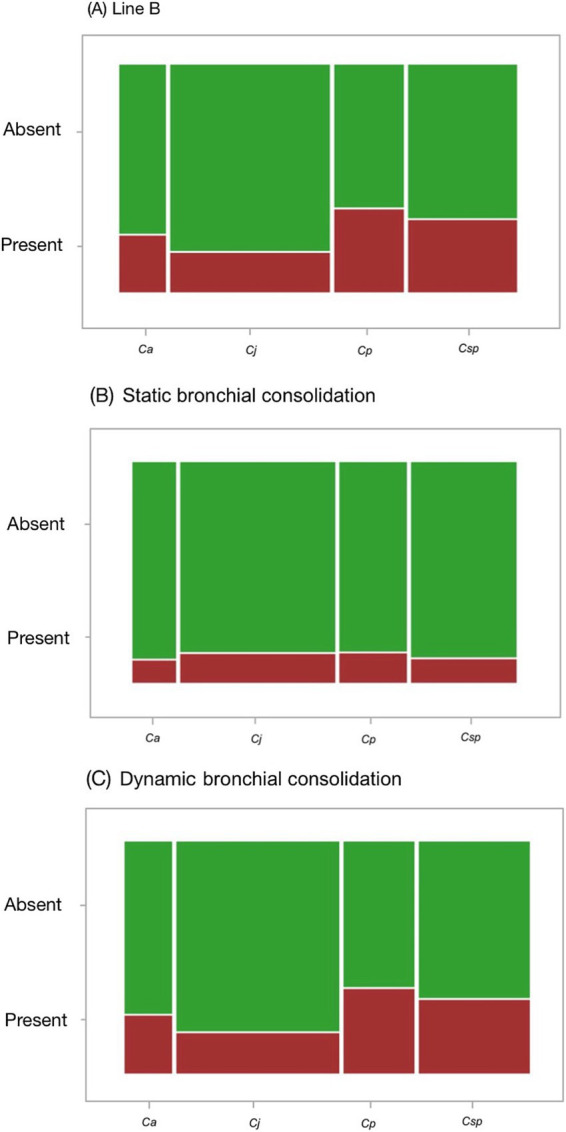
Mosaic graph of the frequency of ultrasound findings from the thorax of neotropical primates of the genus *Callithrix*. The height of the bars represents the relative frequency of ultrasound findings, and the width represents the sampling effort for each species. **(A)** shows the frequency of artefact lines B, in **(B)** the frequency of consolidations with static bronchograms and in **(C)** the frequency of consolidations with dynamic bronchograms. Key: Ca, *Callithrix aurita*; Cj, *Callithrix jacchus*; Cp, *Callithrix penicillata*; Csp, *Callithrix* sp.

Line A was found in all the animals evaluated, while line B was found in 26% of the marmosets (43/166). Static bronchial consolidation (SBC) was seen in 12% of the marmosets (20/166) and dynamic bronchial consolidation (DBC) was seen in 26% of the marmosets (43/166). Table 1 the frequency of these findings among the species analyzed. No significant differences were detected between the marmoset species, regarding the frequencies of line B (*χ*^2^ = 5.309, df = 3, *p* = 0.151), SBC (*χ*^2^ = 0.279, df = 3, *p* = 0.964) and DBC (*χ*^2^ = 4.699, df = 3, *p* = 0.195) (Figures 3, 4).

The occurrence of SBC was heterogeneous in relation to the incidence of line B (*p* = 0.014), such that it was more frequent in animals in which line B was present (22%) than in those in which line A was absent (8%). Similarly, the incidence of SBC was also heterogeneous in relation to the incidence of line A (*p* = 0.005), such that it was less frequent in animals in which line B was present (40%) than in those in which line B was absent (19%). On the other hand, there was no significant heterogeneity regarding the incidence of SBC and DBC (*p* = 1) (13).

The ultrasound findings were not influenced by the sex, age or mass of the animals. The frequency of the main findings was not statistically different between the marmoset species.

Three animals that died from bronchopneumonia, marked by necrotic neutrophils in the bronchial and bronchiolar lumen, had dynamic bronchograms and one of them had associated SBC. These sonographic findings were seen in cases of bacterial suppurative origin and also aspiration origin, this may indicate that dynamic bronchograms may be associated with bronchopneumonia, three animals presented the B-line sonographic finding in association with alveolar oedema and congestion, as reported in dogs and humans, three animals presented SBC associated with a congestive process, and one animal presented DBC associated with a congestive process (Supplementary material).

## 4 Discussion

Until now, there had been no research on use of thoracic ultrasound among non-human primates, which was why it was necessary to make correlations with studies conducted on humans and on dogs and cats.

Pulmonary auscultation in *Callithrix* sp. is difficult to perform because of the sounds emitted by these animals when they are not under sedation. However, thoracic ultrasound can be performed on awake animals that are manually restrained and can provide important pulmonary assessment parameters, the animals in this study with pathologies were submitted to treatment and ultrasound monitoring without sedation.

For dogs and cats, ultrasound and radiography have proved to be satisfactory methods for both detecting and classifying alveolar-interstitial syndrome. These two techniques show topographical conformity of the alterations, although radiography enables greater detection of involvement of the caudal windows and ultrasound enables greater detection of involvement of the cranial windows. Patients with alveolar-interstitial syndrome and respiratory distress from various causes were analyzed. The animals with cardiogenic pulmonary edema showed a diffuse pattern, while those with pneumonia showed a unilateral pattern, which may suggest that use of a complementary diagnostic method may be useful (14).

In the present study, only lung ultrasound was used, and the animals assessed as exhibiting evidence of lung pathological conditions showed focal alterations (B-lines in 24% of the animals studied, consolidation with static bronchogram in 11% of the animals studied and consolidation with static bronchogram in 24% of the animals studied). Occurrence of consolidations with static or dynamic bronchograms was associated with the presence of the B-line artefact, which suggests that these ultrasound findings are influenced by a common physiological or anatomical process.

One question that needs to be considered regarding this finding is that the individuals evaluated did not show any clinical respiratory signs.

During the study period, some of the animals with lobar consolidations detected through ultrasound died and were sent for necropsy. The macroscopic and histopathological findings indicated the presence of suppurative pneumonia.

In a study carried out to detect pneumonia in children, the location where consolidations were most frequently found was the lower regions of the chest (15). In dogs with bacterial pneumonia, the window most affected was the right cranial window, but involvement of the right and left middle windows was also observed. The fragment sign (consolidation) was evident in the left middle window, right middle window and right cranial window (16). In the animals sampled in this study, the presence of consolidations was observed predominantly in the caudal windows, as previously observed and reported in humans.

Pinpimai et al. (17), isolated *Klebsiella pneumoniae* through a phylogenetic study on the tissues of four marmosets with no previous clinical symptoms that had been found dead in their enclosure, with foamy content in the oral cavity. At necropsy, multilobe pneumonia was observed, and microscopy showed mild suppurative pneumonia with pulmonary edema, emphysema and extramedullary hematopoiesis. Rod-shaped bacteria were detected inside the alveoli.

In Brazil, *Klebsiella pneumoniae* was found in the tissues of 11 marmosets that had been kept at an institution in the state of São Paulo, and histological findings compatible with hyperacute septicemia were observed. In the lungs, the presence of interstitial pneumonia and hemorrhage was reported, highlighting the importance of surveillance of this pathogen due to its emerging nature and its ability to produce resistance to antibiotics and the possibility of infecting human and non-human reservoirs (18).

In a retrospective study on pneumopathies in 638 marmosets diagnosed through necropsy, only 39 did not show pulmonary alterations. The most prevalent inflammatory lung disease was interstitial pneumonia, observed in 206 marmosets. The presence of bacteria was minimal in marmosets with interstitial pneumonia, and these were isolated by means of culture: *Escherichia coli*, *Streptococcus* spp., *Erysipelothrix rhusiopathiae*, *Klebsiella pneumoniae* and *Pseudomonas aeruginosa*. Other forms of pneumonia were rare and included lobar pneumonia in 9 animals, suppurative bronchopneumonia in 6 animals and broncho interstitial pneumonia in 2 animals. Lobar pneumonia was observed and divided according to the type of exudate (purulent or fibrinopurulent). In eight out of nine cases of lobar pneumonia and in all suppurative bronchopneumonia, the classifications were moderate to severe and acute to subacute. These conditions were the main causes of illness or death in most cases. *Streptococcus* spp., with or without association with *Bordetella bronchiseptica*, was isolated in the individuals affected by suppurative bronchopneumonia. *Bordetella bronchiseptica* was also isolated in one animal with fibrinopurulent pleuropneumonia. *Enterococcus* spp. and *Klebsiella pneumoniae* ssp. were also isolated. Circulatory disorders such as edema, hemorrhage and hyaline membrane formation were reported (19).

In pneumopathies caused by SARS-CoV in marmosets, multifocal to coalescent interstitial pneumonitis was reported, with mild infiltrate with a high number of neutrophils in the inter-alveolar septa 2 days after infection. On the fourth day after infection, the intestinal infiltrate was predominantly mononuclear, with an increase in the number of alveolar macrophages and exudates. On the seventh day after infection, the inflammatory condition improved, but edema was reported, with irregular distribution (20). Thoracic radiographic monitoring in ventro-dorsal and latero-lateral projections of nine male marmosets infected with the MERS-CoV virus revealed diffuse interstitial infiltration ranging from mild to severe, with greater involvement of the caudal lobes (21).

In a retrospective study on free-ranging *Callithrix* individuals that died due to *Toxoplasma gondii*, histopathological analysis revealed pneumonia with many intra-alveolar foamy macrophages and fibrin deposition. The pneumonia was interstitial and bronchointerstitial, mild to moderate, with or without multifocal areas of necrosis, and with edema and mild to severe alveolar hemorrhage (22). In the present study, the lungs were found to have an intense infiltrate of necrotic neutrophils in the bronchiolar and bronchial lumens, interspersed between bacterial clusters (rods and cocci). Edema, congestion and thickening of the interalveolar septa were also observed, but it was not possible to isolate the agent. In the present study, the animals submitted to post-mortem histopathological analysis with results compatible with pulmonary oedema associated or not with congestion on thoracic ultrasound showed line B, one animal had an associated B-line and static bronchogram. Meanwhile, animals with post-mortem histopathological analysis compatible with suppurative bronchopneumonia and aspiration bronchopneumonia showed associations of dynamic and static bronchograms on ultrasound evaluation.

Among the individuals that presented alterations on examination, 50 had been kept at an institution in the state of São Paulo at which a fire occurred 2 years before the start of the research of the present study (47 out of these 50 were present at the time of the fire), which may have influenced the findings of the examinations. This was the first study using ultrasound to detect pulmonary pathological conditions in non-human primates. Further research is therefore necessary to determine the frequency of lesions and their most likely topographical locations.

Throughout the study, 41 individuals died, of which 19 had pulmonary alterations. These individuals were sent for necropsy and histopathological analysis, which showed that 10 had alterations in the lung tissue, including suppurative pneumonia and pulmonary congestion, which in some cases was associated with oedema. In the sample, 8 individuals showed alterations suggestive of congestion and pulmonary oedema and on ultrasound the B-line artefact was observed in 6 of these individuals, which corroborates findings already reported in humans and domestic animals.

Regarding the 9 animals that showed no alterations on histopathological examination, one hypothesis is that they died between 6 and 18 months after the date of the ultrasound assessment and as there was no serial monitoring, there is no way of determining their evolution. Further research is needed involving other imaging techniques such as radiography and computed tomography, in order to assess the sensitivity and specificity of these different techniques for these species.

Lung ultrasound combined with chest radiography has proved to be a sensitive method for diagnosing small lobar consolidations and for the early detection of pleural effusion, although, some practical limitations in using ultrasound techniques have been reported in their use among humans, including the impossibility of assessing the retro-scapular region, difficulty relating to overlapping structures, need for an experienced operator (23). And the presence of subcutaneous emphysema and dressings, which can compromise the quality of the examination (24).

In neotropical primates, the retro-scapular region has been assessed without difficulty due to the anatomy of these species. However, the operator’s level of experience is fundamental for identifying image alterations, and care must be taken not to confuse the liver parenchyma and presence of gastric contents with alterations in the dorso-caudal pulmonary windows.

## 5 Conclusion

Thoracic ultrasound is a useful tool in the clinical routine for assessing wild animals. It has good applicability due to its low cost, quick execution time and non-invasive nature. It has shown good sensitivity for diagnosing pathological lung conditions. The pulmonary patterns observed are similar to those found in human medicine, in terms of the appearance and localization of the alterations. In this study, it was possible to correlate the frequency of pulmonary consolidations identified through thoracic ultrasound and histopathological findings with occurrences of pneumonia, and to correlate the presence of the B-line artefact, with or without association with pulmonary consolidation, with congestion, which may or may not be associated with oedema in *Callithrix* sp.

## Data Availability

The original contributions presented in the study are included in the article/Supplementary material, further inquiries can be directed to the corresponding author.
