# Supplementary material for: Thoracic ultrasound for diagnosing pneumopathies in neotropical primates
Source: Front Vet Sci. 2024 Dec 17;11:1450104. doi: 10.3389/fvets.2024.1450104 (PMC11687002; doi:10.3389/fvets.2024.1450104)
Supplement: Supplementary file 1 [file Table_1.DOCX]

Supplementary Material

# Supplementary Data

# Supplementary Chart

Chest ultrasound alterations associated with histopathological findings from 10 of the 19 individuals that died

| 26 | DBC - CrR | Lungs: interstitium showing slight vascular congestion, with no evidence of inflammatory changes. Alveoli, bronchioles and bronchi preserved |
| --- | --- | --- |
| 43 | DBC - CrL | Lungs showing marked infiltration of necrotic neutrophils in the bronchiolar and bronchial lumens, interspersed between bacterial clusters (rods and cocci) and necrotic cell debris. The alveoli showed marked peribronchial edema and the interstitium showed vascular congestion. The corresponding morphological diagnosis was suppurative bacterial bronchopneumonia. |
| 46 | SBC - CrL/ DBC - CrR | Lungs with marked infiltration of necrotic neutrophils in the bronchiolar and bronchial lumen, interspersed between coccoid bacterial clusters and necrotic cell debris. The interstices showed marked vascular congestion and thickening of the interalveolar septa, as well as pulmonary alterations, the cardiomyocytes showed viscous nuclei and formed thick muscle fibers (hypertrophy). The morphological diagnosis for this case was suppurative bacterial bronchopneumonia |
| 84 | B' - CrL | Lungs: interstitium showing vascular congestion and multifocal alveolar edema. Bronchioles, bronchi and alveoli preserved, without fluid or cell deposition |
| 98 | SBC - CrR | Lungs: interstitium showing vascular congestion and alveoli with multifocal deposition of homogeneous proteinaceous fluid. Bronchi and bronchioles showing preserved integrity, with no evidence of inflammatory, infectious or proliferative alterations. |
| 99 | DBC - CrL | Lungs: marked multifocal infiltration of necrotic neutrophils in the alveolar, bronchiolar and bronchial lumen, interspersed with fragments compatible with plant fibers, proteinaceous fluid (edema), activated macrophages and red blood cells (hemorrhage). The interstitium showed marked vascular congestion and thickening of the interalveolar septa. |
| 111 | SBC - CrL | Lungs: interstitium, alveoli, bronchi and bronchioles showing preserved integrity of the interalveolar septa, with no evidence of inflammatory, infectious or proliferative changes. Moderate vascular congestion was noted. |
| 136 | B' - thorax L | Lungs: interstitium showing moderate and diffuse vascular hyperemia. Alveoli showing multifocal homogeneous proteinaceous fluid deposition (edema). |
| 149 | SBC - CrR | Lungs: interstitium showing slight vascular congestion and multifocal alveolar edema. Bronchioles, bronchi and alveoli preserved, without fluid or cell deposition. |
| 167 | B' - thorax R/L | Lungs: Macrophages phagocytizing red blood cells, with formation of hemosiderin pigment in the alveoli (‘heart failure cells’), with deposition of homogeneous protein liquid (edema). Vascular congestion was noted in the interstitium. |

Abbreviations: DBC, consolidation with dynamic bronchogram; CrR, right cranial window; SBC, consolidation with static bronchogram; CrL, left cranial window; B', line B; Thorax R, all thoracic windows on the right side; Thorax L, all thoracic windows on the left side; Thorax R/L, all thoracic windows on the right and left sides.

**
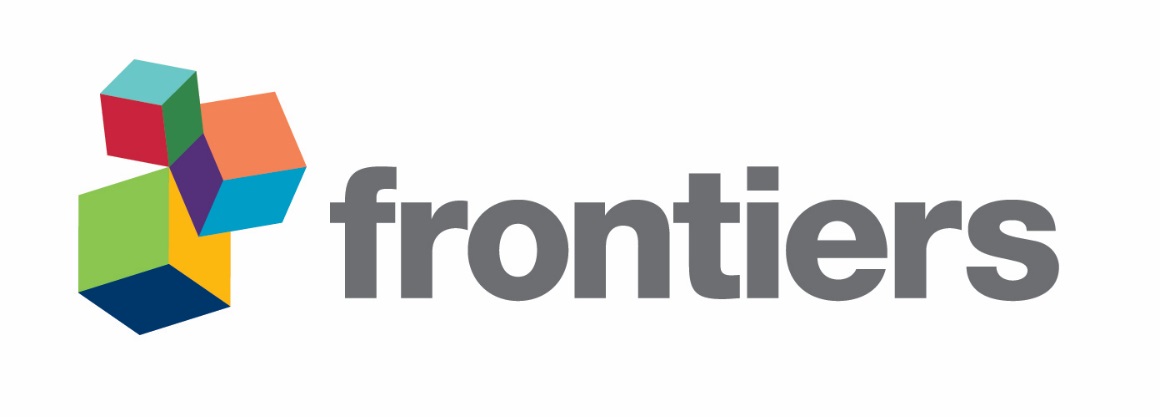
**
